# Cybersecurity Awareness as a Mediating Variable in the Relationship between ICS and AIS in Iraqi State Banks

**DOI:** 10.12688/f1000research.175421.2

**Published:** 2026-05-05

**Authors:** Younis Ahmed Al-Mohammedi, Ahmed S. Tarkh, Hamzah N. Al-Jumaili, Nedhal Aziz Mahdi

**Affiliations:** 1 College of Management and Economics - Department of Accounting, University of Fallujah, Al-Fallujah, Al Anbar Governorate, 31002, Iraq; 2 Internal Audit and Control Department, University of Fallujah, Al-Fallujah, Al Anbar Governorate, 31002, Iraq; 3 College of Administration and Economics, Gilgamesh University, Baghdad, Baghdad, Iraq, 10045, Iraq

**Keywords:** Cybersecurity awareness, internal control, accounting information security, Iraqi government banks.

## Abstract

**Research Objective:**

This research aims to study the relationship between internal control and accounting information security, while exploring the role of cybersecurity awareness as a mediating variable that can contribute to strengthening this relationship.

**Research Significance:**

This research gains its significance from bridging the knowledge gap in accounting literature concerning the integration of internal control systems with information security in the face of cyber challenges.

**Research Methodology and Tools:**

Considering the nature of the problem and the research objectives, this study follows the steps of the descriptive-analytical method. The aim is to determine the role of cybersecurity awareness as a mediating variable in strengthening the relationship between internal control systems and accounting information security. This includes analyzing data and information related to enhancing the importance of cybersecurity awareness in the local environment, both theoretically and empirically. The analysis was conducted through a survey examining the relationship between internal control systems and accounting information security, mediated by cybersecurity awareness among employees at different administrative levels in the banks included in the study sample. The PLS Smart software was used to process and analyze data. The research found that internal control systems are closely linked to the security of accounting information, with the mediating factor being cybersecurity awareness. This finding indicates a positive and significant relationship. This demonstrates the crucial and positive role of control in protecting data when cybersecurity awareness is raised.

**Recommendations:**

The researchers recommend that banks adopt best practices for information security protection and implement strong and clear policies and procedures for cybersecurity awareness.

## 1. Introduction

Information systems, evidence bases, and communication systems have become the main vein of the world of knowledge, industry, finance, business and other sectors, and the security of information and accounting data. Also, its protection from increasing cyber threats has become crucial considering the digital transformations and a significant expansion in the use of accounting information systems, which can lead to substantial losses if ignored. Security, which justifies the need to understand the factors that enhance the effectiveness of ICS by providing adequate data protection. Despite the Department’s keenness and efforts to implement ICS systems (ICS), the increasing cyberattacks and exploitation of technical gaps have shown a gap in the AIS necessitating the extent for which cybersecurity awareness has an effect on strengthening the relationship between ICS and data security, contributing to designing and developing more effective data security policies in organizations.

In this context, cybersecurity awareness has gained special importance. It is considered a variable that may contribute to strengthening the ICS relationship with AIS, through its active role in enabling employees in banking institutions to identify risks and adopt policies that are understood in accordance with sound security practices. The research relies on data collection by designing a questionnaire addressed to the specialized staff and analyzing it to reveal the relationship between the study variables, in a way that contributes to providing scientific and practical recommendations to enhance the AIS from the risks and security gaps in the contemporary environment.

### 1.1 Research problem

The use of public networks such as the Internet is one of the main trends of banking institutions in recent times, which has caused a qualitative leap in the financial and banking services, but it is hardly without discomforts. These institutions have confirmed that the benefits and services that come to them because of the use of the Internet include many risks represented in the growing incidence of financial fraud cases because of the weakness and absence of effective standards and principles that can be relied on for the verification of the identity of customers and customers. Also, they have a steady increase in the Cases of privacy violation in electronic financial and banking operations because these processes involve multiple stages of repeated collection and transmission of information and data. For the foregoing, banking institutions should attach great importance to the AIS to ensure the protection of information from internal and external threats and risks, protecting and maintain confidentiality and privacy from the expected risks because of the spread of hacking and hacking methods and tools on public networks, and to counter and combat information attacks activities.

ICS are important tools enhancing the security of the information that organizations rely on in the face of the threats they are exposed to. Also, the effectiveness of these systems is not enough to face various threats unless their employees have sufficient cybersecurity awareness.

The ICS relationship with AIS has emerged, while exploring the role of cybersecurity awareness as an intermediate variable that can contribute to strengthening this relationship. According to the literature, employees in organizations are the weakest link in the AIS system, even in the presence of effective ICS unless their employees have sufficient awareness and knowledge of cybersecurity risks and methods, protection and prevention. Hence, the main problem of the research emerges, which is represented in the following question:

How are cybersecurity awareness and internal control systems (ICS) associated with the protection of accounting information systems (AIS) among employees of banking institutions in Anbar province? This main question includes:

1. How big is cybersecurity awareness among employees of government banks in Anbar province?
2. How effective is the ICS applied in these banks?
3. How efficient is the AIS in these banks?
4. What does cybersecurity awareness relate to AIS among employees of these banks?
5. What is the relationship between ICS and AIS?
6. Does cybersecurity awareness contribute to enhancing the effectiveness of the ICS to protect the AIS?
7. What is the complementary role of both cybersecurity awareness and ICS in protecting AIS?

### 1.2 The study importance

The study is important because of its effective contribution to bridging the knowledge gap in the accounting literature related to the integration of ICS with information security considering cyber challenges. It highlights the role of cybersecurity awareness as an intermediate variable, an aspect that has rarely been addressed in previous studies in the government context. The research also provides a theoretical framework that can be used in future studies dealing with cybersecurity in the public sector. The research also addresses a real problem facing government banking institutions, namely the weak protection of accounting information because of weak control or the absence of security culture. It helps decision-makers design effective control and awareness policies to protect financial data from cyber threats.

### 1.3 Research objectives

Considering the nature of the problem that the researchers seek to address the main aspects of it to show the role played by cybersecurity awareness as an intermediate variable in the ICS relationship to AIS, the following secondary objectives emerge from this main goal:

1. Identifying the level of cybersecurity awareness among employees of bank branches of Al-Rashid Bank in the cities of Fallujah and Ramadi.
2. Measuring the ICS effectiveness in those banks.
3. Assess AIS in these banks.
4. Analyze the cybersecurity awareness relation to AIS among employees of these banks.
5. Study the ICS relationship with AIS.
6. Reveal the extent to which cybersecurity awareness contributes to enhancing the effectiveness of the ICS to protect the AIS.
7. Identify the complementary role of both cybersecurity awareness and ICS in protecting the AIS among employees in banking institutions in the study sample.

## 2. Second topic/Theoretical aspect of the research

### 2.1 First: Components and elements of the ICS system

“ICS is designing activities and procedures by the board, management and employees of an entity”. It aims to provide a reasonable degree of certainty as to gain the organization’s aims related to asset protection and operational efficiency, the preparation and accuracy of reports, and compliance with the relevant laws and regulations through the procedures and policies adopted for this purpose ( COSO, 2013).

According to Abdullah (2007, 229), ICS is the organizational plan, means of coordination and measures designed by the bank’s management to protect its assets, controlling and auditing accounting data, ensuring its accuracy and reliability, raising productivity efficiency, and encouraging employees for the adherence to the set management policies.

According to Ryabov (2021), the ICS is an integrated system that includes organizational plans designed by the economic unit, which implicitly includes the methods to protect its assets, test the accuracy of its data. It improves its operations and encourages adherence to the administrative policies set by it.

So, ICS ensures that banking institutions gain their strategic aims by carrying out their financial and operational operations efficiently and effectively, in addition to contributing to preserving the resources available to the economic unit from damage, loss, misuse and other non-ideal conditions that may occur and affect those units.

In order for the ICS to be effective, it should be strengthened by a set of elements and elements, including but not limited to the existence of a flexible and clear organizational plan that is clearly understood and applied, and the bank’s accounting system has to be sound, clear and simple to ensure effective ICS at all stages of the banking business, by making the accounting cycle and ways of documenting it clear. It must also be ensured that tasks should be distributed and segregated among employees to reduce the likelihood of intentional and unintentional deviations and irregularities affecting financial reporting. It is essential to select qualified and experienced staff, especially those in charge of the ICS system, to ensure that performance is controlled. Also, all levels of staff from top to bottom are obliged to follow the set goals and plans, and to avoid deviations at all levels. Thus, mechanisms should be put in place to address deviations if they occur by checking them and acting Corrective Appropriateness ( Qourin et al., 2019).

Banks in government institutions must provide protection against any type of cyber-attacks that they are expected to face during the implementation of their operations, so they are in dire need of cybersecurity ( Goutam & Verman: 2015) involving functions related to the management of cyber risks related to the cyber environment. It also aims to provide the optimal basic requirements and in accordance with the standards and practices set for this purpose, whether those related to the confidentiality of information or those related to ensuring the integrity and integrity of information for reducing cyber risks to technological and information assets for all organizations, whether the threats are exposed to them are internal or external.

Wolden et al. (2015) believed that cybersecurity goals are achieved through key axes for maintaining information security:

1- Technology: This axis includes all the tools and techniques used to protect programs from potential cyber threats, such as using protection software to support and enhance the security of electronic systems against cyber threats.
2- Organizations and Individuals: This axis includes all entities and individuals using information and electronic systems, as information security requires collective cooperation by all members of the organization.
3- Activities and Operations: This theme includes the methods used by government banks in employing personnel and technologies that limit or prevent cyberattacks. Through it, the ICS relationship to cybersecurity is manifested.

According to Bozkus & Caliyurt (2018), ICS and cybersecurity determine banking environment, as institutions in general, and banks in particular, have depended on electronic technologies and applications in using their various activities and processes. This increased adoption has led to their high exposure to breaches and security. An ICS is also essential to a cybersecurity strategy to verify the availability of security and protection in the system contributing to identifying vulnerabilities in systems and applications by estimating security risks, discovering vulnerabilities and flaws in system settings. It ensures that these vulnerabilities are addressed appropriately. In addition, it detects cyber-attacks to determine whether the system has been compromised or has been exposed to previous breaches and attacks and an ICS to analyze system logs and identify any unauthorized intrusions or exploits.

The ICS also contributes to raising awareness and self-security among bank employees, as it highlights existing security vulnerabilities and identifies the necessary actions to avoid them and minimize their effects. This, in turn, enhances the ability of employees to monitor, detect, and prevent cyber threats ( Stevens et al., 2020).

The ICS is one of the essential measures that contribute to enhancing cybersecurity in banking operations, by helping to detect and protect sensitive data and financial and personal information of customers within the bank, by monitoring security threats and cyber breaches through systems monitoring and data analysis. It also contributes to assessing the implementation of security policies and procedures and ensuring their compliance with relevant local and international standards and legislation.

Furthermore, the ICS ensures the Bank’s compliance with cybersecurity regulations by examining various records and reports, especially those related to the implementation of Cloud ERP Systems, where it plays a key role in identifying weaknesses in the system and recommending the required corrective actions This will enhance the cybersecurity of the bank, which helps in making the appropriate decisions within the specified timings to support and enhance the security of banking operations ( Huseynov et al., 2020; Shamsuddin et al., 2018).

### 2.2 Second: The concept, benefits and motivations of spreading the culture of cybersecurity awareness

Cybersecurity is a critical factor in ensuring the integrity of cloud ERP systems and protecting them from multiple cyber threats, through the development of security procedures and policies ( Jamm’e and Alash, 2021). The adoption of specialized protection systems, and the implementation of various technologies are directly related to encryption and advanced protection, and periodic updates ( Ben Alqama and Saahi, 2019). Other requirements are also required related to strengthening collaborative relationships between technical service providers, government entities, security institutions, and banks to reduce cyber risks and ensure the security of accounting data. It also emphasizes promoting and raising awareness among users of the need to follow appropriate security measures to protect their financial and personal data, and to provide bank employees with the elements of competence and technical knowledge in the field of information technology to enhance and improve cybersecurity ( Despotović et al., 2023).

As the online world becomes more interconnected, enhancing cybersecurity awareness has become a necessity for every user, cybersecurity awareness is the cornerstone of having the right defenses against increasingly complex and pervasive cyber threats ( George, George, & Baskar, 2023). People armed with information security awareness understand the importance of data security Whatever their nature, including financial, personal, and corporate data. They are also less likely to fall victim to fraud or inadvertent innuendo and disclosure of important information about the organization.

Having cybersecurity awareness helps to identify potential risks such as malware, phishing, and social engineering fraud early ( Sharma & Thapa, 2023). Conscious behavior also plays a pivotal role in early detection of risks and timely preventive measures, which significantly enhances the overall cybersecurity posture.

Cybersecurity practices represent the preventive and proactive methods adopted to protect the unit’s assets, focusing on identifying expected risks and potential threats and seeking to minimize their effects ( Stransact, n.d.).

This approach is essential to counter complex threats to organizations, emphasize the confidentiality of information, and enhance organizational resilience in the face of the ever-changing environment of cyber threats. Effective cybersecurity practices enable organizations and individuals to remain vigilant and always ready for the purpose of making strategic and critical decisions as well as adopting measures that reduce the risk of cyber-attacks. The magnitude of the growing cyber risks and threats ( Thakur, 2024; Efijemue et al., n.d).

The main objective of promoting cybersecurity awareness and practice in organizations is to train other employees in its importance and to consolidate the organization’s commitment to protecting sensitive information among its employees. The growth and increase in cybersecurity awareness enhances employees’ skills and provides them with the knowledge necessary to address security challenges, including the initiation phase of incident response processes, followed by communication with relevant parties. This is followed by the process of promoting and supporting recovery efforts ( Al-Hawamleh, 2024).

The importance of training employees on cybersecurity awareness in banking institutions cannot be underestimated. In a world where cyber risks and threats are constantly growing in complexity and pervasive, it is imperative that organizations equip their employees with the knowledge and skills necessary to recognize and respond to anticipated risks. This training aims to empower years in organizations to become a firewall against cyberattacks, protecting both their interests and customers’ trust in them.

The benefits of cybersecurity awareness training are not limited to just preventing data breaches and attacks but extend beyond that. Those who are informed and vigilant can foster and spread a culture of security awareness within the organization. Those who can understand potential risks and have sufficient qualifications to follow best practices have a greater ability to make sound decisions when dealing with sensitive information. They organization’s devices, reducing potential accidents and limiting anticipated losses, legal liabilities and reputational damage.

The need to train employees on cybersecurity awareness has become even greater as companies embrace digital transformation and implement remote work policies, as employees access the organization’s data and systems from multiple locations and devices, the cybercriminals’ attack space expands. Well-designed training programs can enhance the knowledge of employees of organizations about the specific risks of remote work. Providing them with all the necessary knowledge to secure their networks and home devices effectively and efficiently.

Cybersecurity awareness training is an initiative-taking and necessary investment for organizations that aim to protect their digital assets and maintain their competitive advantage in today’s era. By equipping employees with the knowledge and skills that help identify and respond to cyber threats, organizations can reduce or reduce the likelihood of successful fraudulent attacks and protect their resources and maintain their reputation in the competitive market.

### 2.3 Third: The concept and relationship between AIS and cybersecurity awareness

A study submitted by ( ASIF, 2023) for information security refers to the strategic methods and techniques used by the organization aimed at protecting and securing confidential information from unauthorized access by hackers who engage in activities of criminal nature online or carry out acts of sabotage or illegal weakening. Also, information security can be described as practices aimed at Enhance information security and stand up to unauthorized access.

As information is one of the key assets of organizations, it requires the concerted efforts of all through the participation of diverse groups of stakeholders such as ICT experts, information security professionals, management, staff, and office managers who are the lifeblood and custodians of information in the organization.

Lack of awareness of cyber risks and required security practices is a major factor that leads to vulnerabilities. Hence, ongoing security awareness training plays a big role in educating workers about common and potential risks such as phishing and social engineering attacks, resulting in a reduced likelihood of occurrence Human errors that lead to security breaches ( Andersson, Bjursell, & Palm, 2023).

While organizations invest significant resources in advanced tools and technologies that enhance cybersecurity, they alone are not enough to provide comprehensive protection against threats. Workers are often the weakest link in the information security chain and may fall victim to social engineering tact ICS, opening malicious mail attachments, or disclosing and publishing Unintentionally sensitive information. Therefore, it is essential to add to their security strategies that organizations prioritize cybersecurity awareness training.

As detailed above, security risks cannot be eliminated even with the use and adoption of control and preventive measures and procedures, but they can be significantly reduced and scaled down through the creation and updating of targeted plans for the purpose of responding to incidents and confronting breaches or data leaks quickly and effectively.

In the context of cybersecurity awareness and information security, there is an integral relationship between them in organizational processes and activities. The more attention employees pay to cybersecurity and its practices, the safer the organization’s environment becomes. In turn, a culture of cybersecurity awareness can be promoted and disseminated through effective information security processes. Therefore, there is a mutually beneficial relationship between the awareness and practices of office managers in the field of cybersecurity and information security, where they contribute These measures promote a culture of cybersecurity awareness, while workers’ interest in cybersecurity promotes building a safer work environment. Therefore, the two years should have a firm awareness of cybersecurity considering the escalating risks and threats of cybercrime and the digital transformation that the contemporary environment is witnessing, with the aim of protecting sensitive data in organizations.

## 3. Methodology

### 3.1 Literature review and hypothesis development

**1. Internal Control and Accounting Information Security**

Mishra and Dhillon (2008) aimed to show the role of ICS in the effectiveness of AIS in general. The study provided a theoretical framework for the basic objectives and means of ICS in the light of AIS Evidence were gathered through in-depth interviews with 52 IT managers that included questions about their assessment of ICS. The study identified 68 objectives, organized into 25 groups, the findings of which serve as a basis for providing more theoretical frameworks in the field of information security governance. The objectives also help define initiatives and policies related to governance.

Al-Sorihi et al. (2025) examined and tested ICS as a mediating variable between IT risks and AIS in telecommunications companies in the Arab Republic. To achieve study aim, the comprehensive survey method was used using the questionnaire as a data collection tool, and 356 questionnaires were distributed, of which 218 questionnaires were used to analyze the data. It was concluded that information technology risks have a negative effect on the AIS systems by 47.6% before the mediation of internal control, and that technology risks have a negative impact on the AIS systems after ICS mediation by 25.3%, where part of the impact is transmitted through internal control. Constantly updating them, paying attention to integrity, confidentiality, and information, keeping pace with technological developments, and effectively monitoring the implementation of the security policy contributes to raising the information security level and reducing the negative impact of information technology risks.

Abdel-Jaber (2013) identified the validity of ICS procedures in Jordanian industrial companies by the electronic accounting information systems in the reduction of their information security risks. In addition, the obstacles impact this effectiveness by the identification of three types of risks that threaten the AIS systems, such as network hacking risks, social engineering risks, and malware risks. For the achievement of the study objectives, a questionnaire was given to the study sample consisting of thirty industrial companies operating in the Hashemite Kingdom of Jordan using accounting information systems. For the data analysis, the statistical methods represented by arithmetic averages, standard deviations, Kruskal Wallace test, Mann and Wintney test, and the test was used. The ICS procedures provided information security in Jordanian industrial companies through its prevention, detection, and correction, the researcher recommended the need for the department to carry out ICS activities on information security through the provision of qualified cadres, and motivating employees’ industrial companies for obtaining relevant professional certificates.

Based on the results of previous studies, the following hypothesis was developed:

H1: There is no statistically significant effect of internal control on accounting information security in government banks in Anbar Governorate.

**2. Internal Control and Cybersecurity Awareness**

Mohamed (2024) shed light on the role played by SAIs in evaluating information systems and cybersecurity, with a focus on the challenges facing audit institutions and their importance in enhancing governance and transparency. The researcher adopted the inductive analytical method, where a theoretical framework was presented for the evaluation of information systems and cybersecurity. The study found out importance for a comprehensive understanding of the basic concepts of information systems control and cybersecurity and the identification of the main objectives of information technology audit. The study also found that strengthening control aspects of information systems and cybersecurity not only contributes to achieving transparency and efficiency but also contributes to enhancing trust between the public and institutions making sure the business continuity and protection of the sensitive data from cyber-attacks.

Mansour (2021) Showed that cybersecurity is important through its effect on ICS and the economic unit value by the adoption of the Information Technology Governance Framework, (COBIT5). The descriptive-analytical approach was used in the data collection along with a questionnaire that included a set of questions divided into eight axes that reflect the research requirements, using Google Forms, and Steven’s equation. Thompson was adopted in determining the size of the sample (98 auditors and accountants) working in higher education and scientific research. There was and general agreement on the relationship between the dimensions and requirements of cybersecurity on the modern frameworks of ICS COBIT5 and the value of economic unity. There was a need for the economic unit to adopt effective means of continuous evaluation of ICS to maintain information security by adopting COBIT5 by integrating procedures and characteristic ICS according to modern frameworks for avoiding the means of penetrating electronic systems and manipulating their information.

Based on the results of previous studies, the following hypothesis was developed:

H2: There is no statistically significant effect of internal control on cybersecurity awareness in government banks in Anbar Governorate.

**3. Internal control, cybersecurity awareness, and accounting information security**

Al-Qusayr (2024) designed and developed an information technology governance model to reduce cyber risks and enhance the AIS in Libyan public institutions. The researcher followed the descriptive-analytical approach by reviewing previous studies, reports, models, best practices and related literature, and the study reached a conclusion that the most important pillars of the proposed model were: governance, the Information Technology Governance Committee and its mechanisms, and the independence of the internal auditor, with an emphasis on risk management, active and continuous control, transparency and accountability.

Based on the results of previous studies, the following hypothesis was developed:

H3: There is no statistically significant effect of cybersecurity awareness on accounting information security in government banks in Anbar Governorate.

H4: There is no statistically significant effect of internal control on accounting information security when cybersecurity awareness is present as a mediating variable.

### 3.2 Informed consent

All survey participants were adults of sound mind and legal capacity; no minors were included. They are employees in various administrative positions at Al-Rasheed Bank and provided their voluntary verbal consent before participating. This was due to the nature of the study, the limited risks involved, and the fact that the research procedures did not pertain to health or medical issues. The participants’ social and cultural context was also taken into consideration. They were informed of the study’s purpose and nature and assured that their responses did not include personal information or any statements related to racial discrimination. Instead, they provided data related to cybersecurity, the protection of accounting information, and internal controls.

### 3.3 Ethical considerations

The information contained in this research is complete and accurate and does not violate internationally recognized laws and ethics, as approved by the Research Ethics Committee at the University of Fallujah. This research has received approval from the Research Ethics Committee of the College of Administration and Economics at the University of Fallujah, under number [UOF.HUM.2025.001].

### 3.4 Population and sampling procedure

The research included a community represented by the government banking sector (Rashid Bank), where a sample was chosen from this community represented by employees and workers in the departments related to the nature of the studied variables, especially the accounts and financial affairs departments and the departments related to information security in this bank in its branches in the cities of Fallujah and Ramadi.

**3.4.1 Sample Description and Demographic Characteristics**

The characteristics of the sample covered by this study include Age, Academic qualification, Scientific specialization, Job title, and Years of experience. Table 1 shows the detailed distribution of the demographic characteristics of the participants. The results of the descriptive analysis of the demographic characteristics of the research sample of 50 individuals show that the sample is characterized by young professionals with high academic qualifications. The sample was concentrated in the age group of 35 years or younger, and 80% of them hold a bachelor’s or master’s degree. There is also a very high concentration of financial and administrative specializations, with 40% of the sample majoring in accounting and 16% in business administration. In terms of experience, the sample exhibits a good balance between intermediate and advanced experience, with 46% of the sample having between 15 and 20 years of experience, and 24% having between 16 and 20 years of experience, creating a good balance between new and experienced talent. Job titles show great diversity, with a good distribution between technical and administrative positions, and a large “other” category comprising 42% of the sample.

**Table 1.** Demographic characteristics of the sample.

Variable	Category	Category number of individuals	Percentage (%)
Age	30 years younger	10	20%
	31-35 years	15	30%
	36-40 years	9	18%
	41-45 years	9	18%
	46-50 years	6	12%
	Over 50 years	1	2%
Academic qualification	Bachelor’s	24	48%
	Higher Diploma	3	6%
	Master’s	16	32%
	Doctorate	4	8%
	Other (specify)	3	6%
Scientific specialization	Accounting	20	40%
	Business Administration	8	16%
	Economics	2	4%
	Public Administration	2	4%
	Financial and Banking Studies	9	18%
	Other (specify)	9	18%
Job title	Department Manager	10	20%
	Auditor	8	16%
	Accountant	11	22%
	Other (specify)	21	42%
Years of experience	Under 5 years	15	30%
	6-10 years	10	20%
	11-15 years	13	26%
	16-20 years	8	16%
	Over 20 years	4	8%
Total		50	

### 3.5 Developing the study tool

The research tool is based on a set of variables that represent the conceptual framework of the research. The research tool includes three main variables: The first variable is the independent variable, which represents internal control, which was measured through five main indicators: the control environment; control activities; follow-up; risk assessment; information and communication. This variable includes (25 items). The second variable is the dependent variable, which represents accounting information security. It was measured as a standardized variable, including (8 items). The third variable is the mediating variable, which is cybersecurity awareness. It was also measured as a standardized variable, through (8 items). Details of the variables, their dimensions, and the number of paragraphs is presented in Table 2.

**Table 2.** Research variables.

Variable type	Variable	Abbreviation	Dimensions/Components	No. of paragraphs
**Independent Variable**	Internal Control	IC	Control Environment (CE)	5
			Control Activities (CA)	5
			Monitoring (MON)	5
			Risk Assessment (RA)	5
			Information and Communication (IC)	5
**Dependent Variable**	Accounting Information Security	AIS	(As a unified concept)	8
**Mediating Variable**	Cybersecurity Awareness	CAW	(As a unified concept)	8

### 3.6 Data collection procedures

The research relied on a questionnaire as the primary means of collecting data related to the research variables. The questionnaires were distributed directly to a group of officials and employees specializing in internal control and cybersecurity, employees working on accounting data processing, and financial report preparers at the Rashid Bank in its two branches in Fallujah and Anbar. The aim was to ensure that the respondents were aware of the nature and accuracy of the questions. A total of 50 questionnaires were distributed, all of which were returned, reflecting a high degree of seriousness and cooperation from the participants. The process of distributing and retrieving the questionnaires took a month and a half, including filtering and organizing the data until it was ready for statistical analysis.

### 3.7 Analytical framework (Quantitative)

To test and discuss hypotheses and evaluate the relationship between variables, quantitative analysis was used, relying on the statistical software SmartPLS, which specializes in structural equation modeling (SEM). This modeling is typically used to explain multiple statistical relationships simultaneously through visualization and model validation. Complex models can be easily discussed using this technique. It is an extension of traditional linear modeling techniques, such as multiple regression analysis and analysis of variance (ANOVA), which are essential requirements for learning structural equation modeling. This modeling adopts a confirmatory approach rather than an exploratory approach. Thus, we will model the impact of internal control on accounting information security, both directly and indirectly through cybersecurity awareness.

### 3.8 Model specifications

The mathematical model was designed to measure the effect of the independent variable, internal control (IC), on the dependent variable, accounting information security (AIS). An intermediate variable, cybersecurity awareness, was also introduced to examine whether this awareness conveys (or mediates) the effect of internal control on information security. For modeling purposes, internal control is a multidimensional variable consisting of its five components (control environment, control activities, monitoring, risk assessment, and information and communication), while the other variables include multiple indicators that serve as the basis for measuring these variables.

To ensure the accuracy of the results and obtain reliable confidence intervals, the model was first tested using the PLS-SEM algorithm. The purpose is to verify the model’s reliability and validity. Bootstrapping will then be used as the primary analysis tool within the structural equation modeling program.

**Figure 1.**
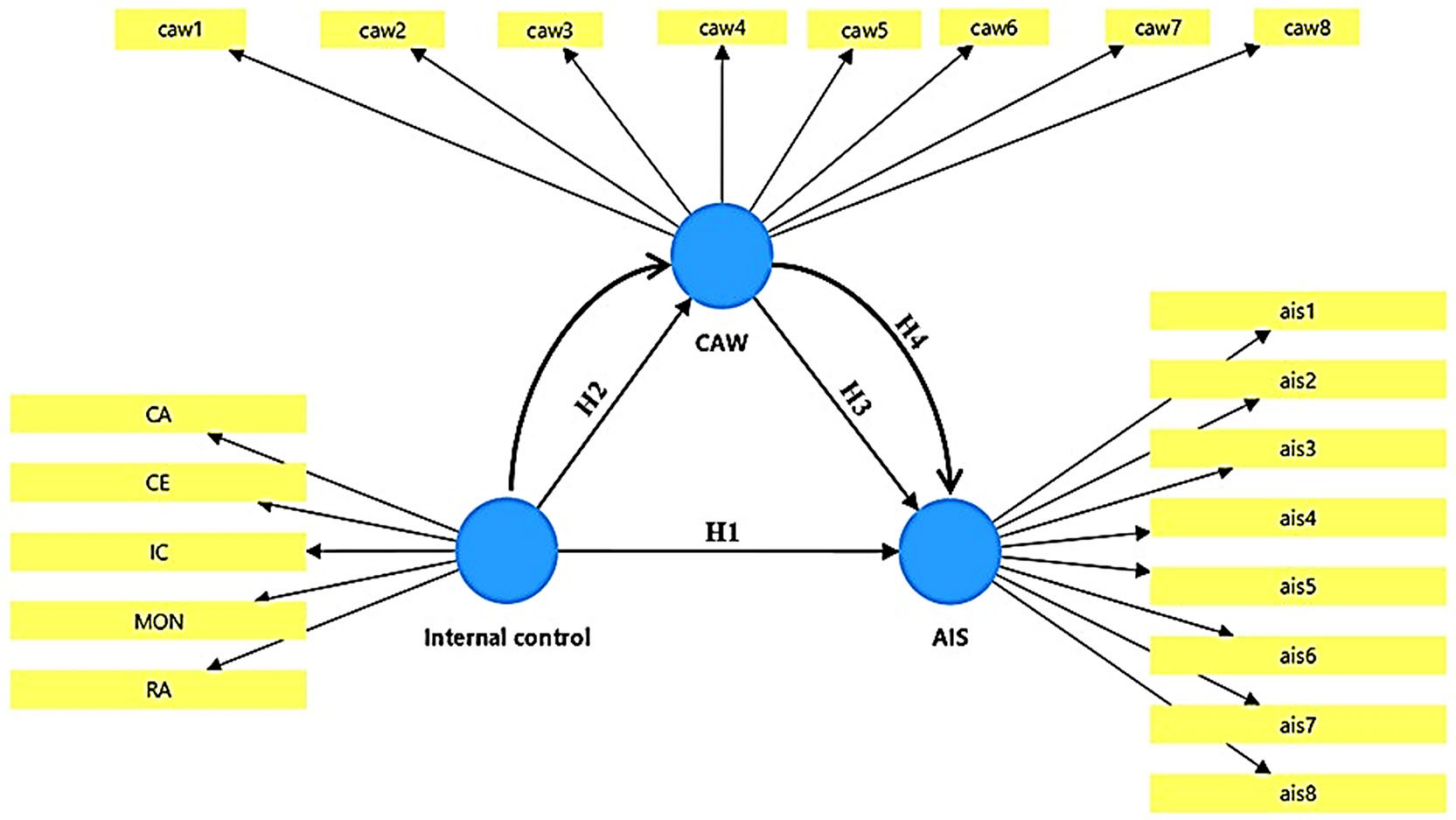
Conceptual model.

The structural structure of the mathematical model can be seen in Figure 1.

Below is the estimation of the mathematical model based on the structured hypothetical relationships, which will be statistically examined:

$$\mathrm{AIS}=a_{0}+\beta_{1}\mathrm{IC}+\varepsilon$$ (1)

$$\mathrm{CAW}=a_{0}+\beta_{2}\mathrm{IC}+\varepsilon$$ (2)

$$\mathrm{AIS}=a_{0}+\beta_{1}\mathrm{IC}+\beta_{3}\mathrm{CAW}+\varepsilon$$ (3)

## 4-Evaluation of the mathematical model (Reliability and internal consistency)

To evaluate reliability and internal consistency, the Loadings test, Cronbach’s alpha coefficient and composite reliability rho_c were used. The results showed that the reliability and internal consistency analysis of the mathematical model adopted in the research showed that the measurement tools were characterized by a very high level of reliability and validity, indicating the quality of the statistical design and the integrity and validity of the data for statistical analysis. The results shown in Table 3 related to the Cronbach’s alpha test showed that the reliability values exceeded the acceptable statistical limit of (0.70) for all variables, ranging between (0.886-0.928). This indicates a high degree of internal consistency between the items of each variable, and that all items measure the same dimension with a high degree of accuracy and consistency. The results of the composite reliability test also confirmed very high values, exceeding (0.90) for all variables, which confirms that the indicators used measure and interpret the latent dimensions in a stable and consistent manner. The results of the weighted average variance (AVE) showed that all values were greater than 0.50, which is the minimum statistically acceptable value. This confirms that the model has convergent validity, meaning that the items can explain most of the variance occurring in the latent variables. This confirms that the model accurately measures what it is supposed to measure; and P-values reached 0.000, which is a strong indication that the correlation coefficients between the items and the latent variables are real and not the result of statistical chance (see Table 3).

**Table 3.** Reliability and internal consistency.

	Cronbach’s alpha	Composite reliability (rho_a)	Composite reliability (rho_c)	Average variance extracted (AVE)	P values
AIS	0.925	0.932	0.939	0.658	0.000
CAW	0.886	0.891	0.910	0.560	0.000
Internal control	0.928	0.931	0.946	0.778	0.000

**Figure 2.**
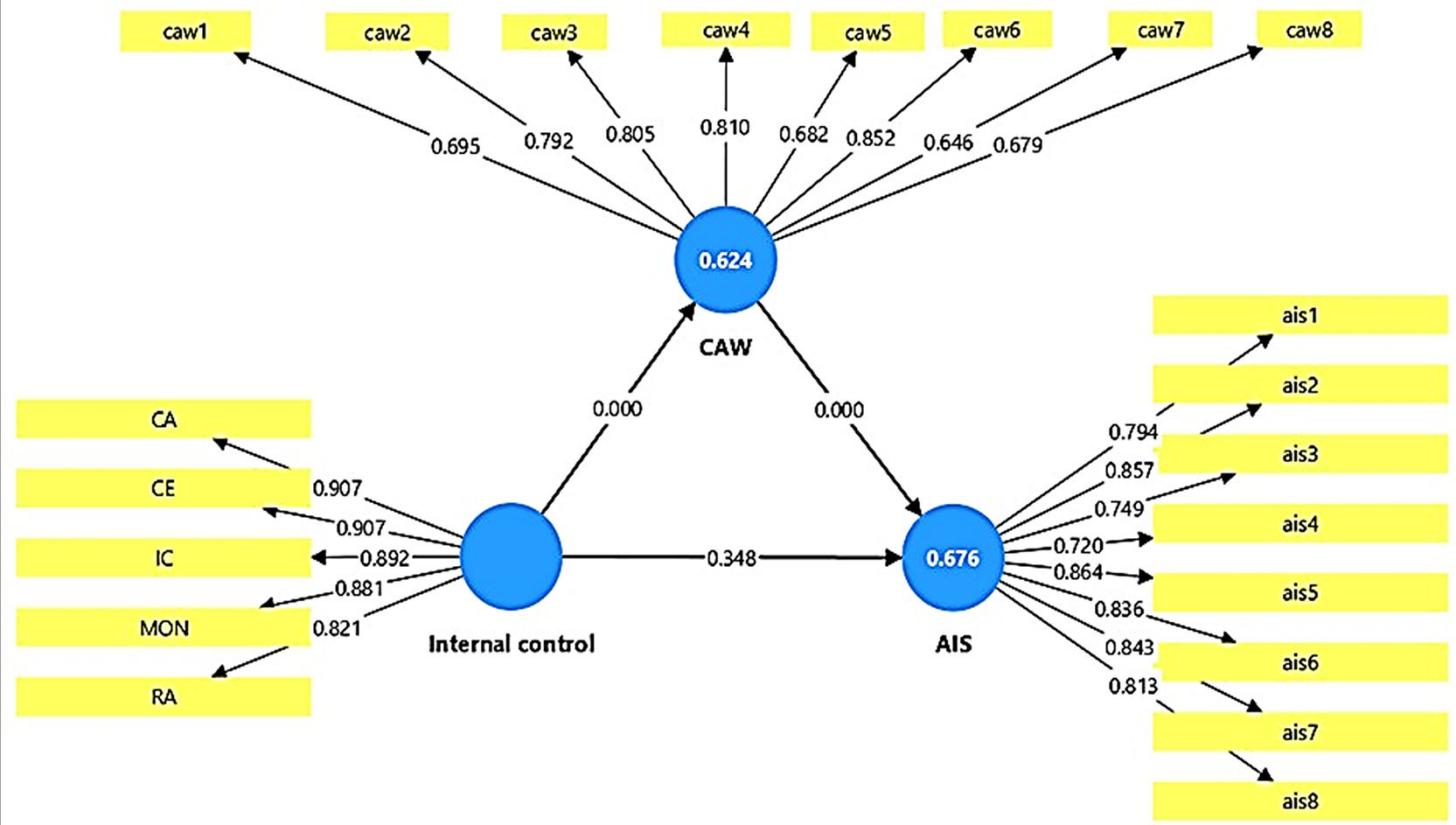
Outer loading and R ^2^ in PLS-SEM algorithm.

The results of the outer loadings test show that all items related to the study variables had positive and significant loadings, exceeding the standard value level of 0.70 (see Table 4). This indicates that each item of the variables is associated with the variable to which it belongs. Furthermore, items with loadings slightly lower than this level are still acceptable due to their high statistical significance. Thus, it can be concluded that the proposed model possesses a high degree of stability and validity, and that the research measurement tools used meet the statistical quality requirements required by partial structural equation models (PLS-SEM). These results confirm the possibility of relying on current research data to test causal relationships between variables with high confidence, giving the model explanatory power and high scientific credibility. **See** Figure 2 To test the discriminator validity of the study model, the Heterotrait-Monotrait Ratio (HTMT) was used. The results of the discriminator validity test showed that all values fall within the statistically acceptable levels (see Table 5). All variable results fall between (0.839-0.871), which is less than the recommended maximum (0.90). These statistics confirm that each variable in the model is distinct from the other variables, meaning that there is a clear distinction between the measured concepts and no overlap between them. The results also showed that the confidence interval (2.5% - 97.5%) for all relationships did not exceed the critical value (1.00), which enhances the discriminator validity of the model and confirms that each dimension measures a different and specific aspect of the phenomenon studied. Thus, the discriminator validity of the latent model is well achieved.

**Table 4.** Outer loadings results.

	AIS	CAW	Internal control	P values
CA			0.907	0.000
CE			0.907	0.000
IC			0.892	0.000
MON			0.881	0.000
RA			0.821	0.000
ais1	0.794			0.000
ais2	0.857			0.000
ais3	0.749			0.000
ais4	0.720			0.000
ais5	0.864			0.000
ais6	0.836			0.000
ais7	0.843			0.000
ais8	0.813			0.000
caw1		0.695		0.000
caw2		0.792		0.000
caw3		0.805		0.000
caw4		0.810		0.000
caw5		0.682		0.000
caw6		0.852		0.000
caw7		0.646		0.000
caw8		0.679		0.000

**Table 5.** Discriminant validity (Heterotrait-Monotrait Ratio -HTMT).

	Original sample (O)	Sample mean (M)	Bias	2.5%	97.5%
CAW<-> AIS	0.850	0.866	0.015	0.655	0.998
Internal control <-> AIS	0.839	0.844	0.005	0.738	0.908
Internal control <-> CAW	0.871	0.871	0.000	0.743	0.943

## 5- Evaluation of the structural model

### 5-1 Coefficient of determination and explanatory power of the model (R2)

The results of the adjusted R ^2^ indicate that the explanatory power of the developed model is large and solid, as it explains a large proportion of the variance in the dependent variable (see Table 6). The adjusted R ^2^ value for the AIS variable reached 0.676, meaning that approximately 68% of the variation in AIS in the studied government banks can be explained by the internal control variable and CAW variable. Similarly, for the CAW variable, the adjusted R ^2^ value reached 0.624, meaning that the model explains approximately 62% of the variance in the cyber awareness variable due to internal control. The above results indicate a high explanatory power and demonstrate the effectiveness of the independent variables in explaining the variance in the accounting information systems in the studied government banks. The T-statistics results also showed values of 7.445 and 8.991, with a significance level of P = 0.000. These values confirm that the relationships between the variables are truly statistically significant and are not caused by chance. This indicates that the model has very good explanatory power, and that the variables used represent realistic and influential interactions within the theoretical framework of the research, which enhances the strength and stability of the results (see Figure 2).

**Table 6.** Coefficient of determination R2 adjusted.

	R-square	R-square adjusted	T statistics (|O/STDEV|)	P values
AIS	0.689	0.676	7.445	0.000
CAW	0.632	0.624	8.991	0.000

To test multicollinearity between independent variables, we relied on the results of Collinearity Statistics (VIF) (see Table 7). The VIF value for both the CAW path to AIS and the Internal Control path to AIS was approximately 2.716, which is well below the critical threshold of 5. These results indicate that each variable independently predicts the interpretation of the dependent variable, without significantly affecting the other. The relationship between internal control and cyber awareness showed a VIF value of 1.000, which is completely ideal and indicates the absence of any linear correlation between them. These results indicate that the model enjoys a high degree of independence between the explanatory (independent) variables, and that the estimated causal relationships reflect the true effects of the variables without bias resulting from statistical interference, enhancing the reliability of the estimates and the quality of the results of the structural model.

**Table 7.** Inner model collinearity statistics (VIF).

	Original sample (O)	Sample mean (M)	2.5%	97.5%
CAW -> AIS	2.716	2.920	1.982	4.230
Internal control -> AIS	2.716	2.920	1.982	4.230
Internal control -> CAW	1.000	1.000	1.000	1.000

### 5-2 Hypothesis testing (Path analysis)

In hypothesis testing, bootstrapping was used, which is the second step in model analysis using the PLS-SEM method. It is used to test the significance of paths and causal relationships between variables in the internal model. Bootstrapping was performed with a power of 5,000 samples to estimate the standard error and extract statistical values (T-values and P-values) that determine the significance of the hypothesized relationships between variables and determine the significance and strength of the relationship using paths between the studied variables.

**Figure 3.**
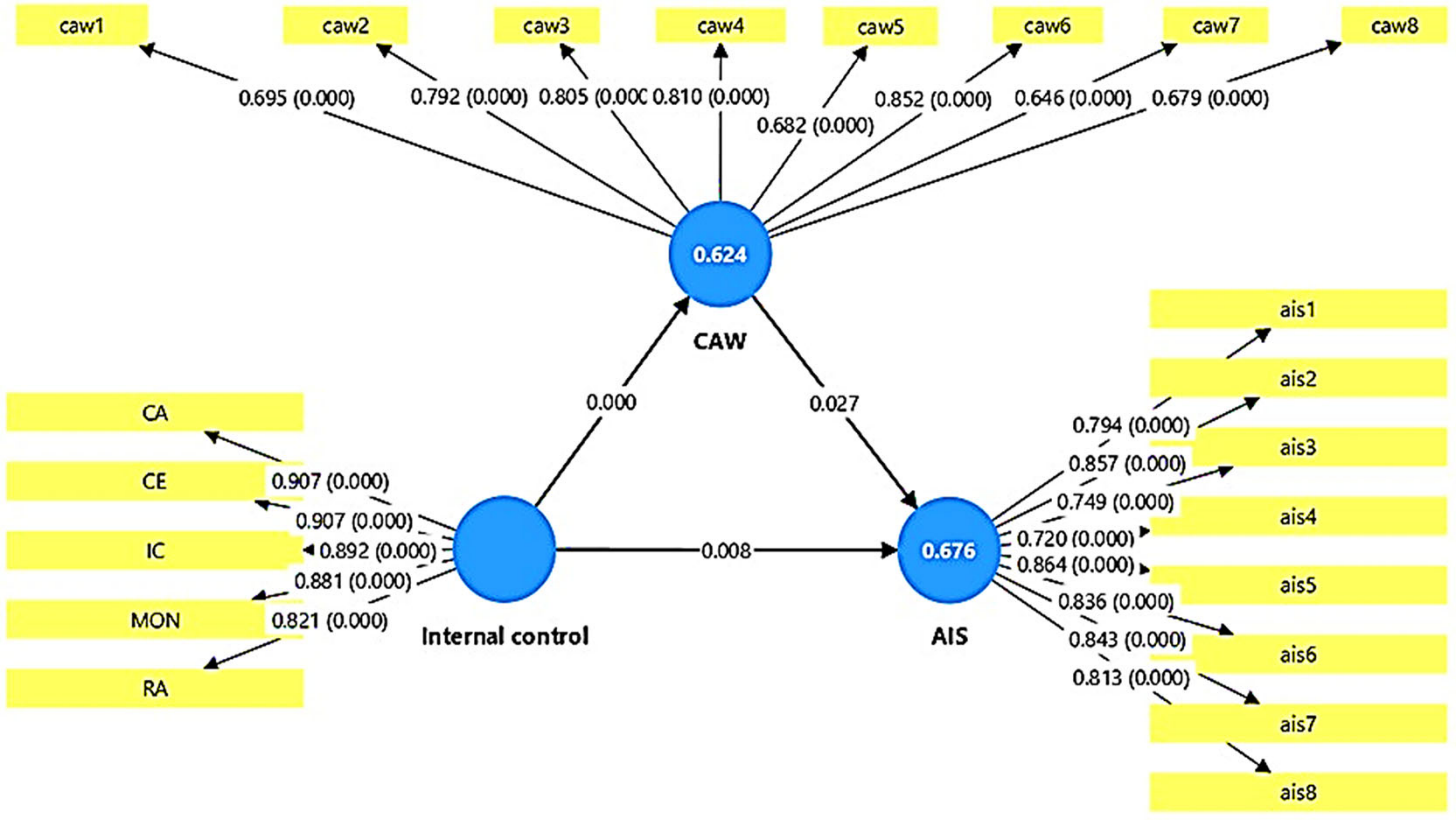
Direct and indirect effects results.

The results of the statistical analysis presented in Tables 8- 10 and Figure 3 showed that all the hypothesized relationships in the statistical model achieved significant significance at the level of (0.05), which reflects the strength of the internal model and its predictive validity. When presenting the detailed results shown in Table (9) related to the direct impact between the variables, these results indicate the presence of a direct and significant positive impact of the internal control variable (IC) on accounting information security (AIS), as the original sample value reached (O = 0.786) with a high t-statistic value (T = 19.001) and a probability value (P = 0.000), which indicates that the internal control variable associated with significantly to enhancing accounting information security within government banks in Anbar Governorate. These results do not support hypothesis (H1), which states that there is no impact of internal control on accounting information security. The results also showed that internal control also has a statistically significant, positive effect at a confidence level of less than 0.005 on cybersecurity awareness (CAW), with an effect value of (O = 0.795, T = 18.447, P = 0.000). This indicates that activating internal control elements contributes to raising the level of awareness and commitment to cybersecurity among employees of the banks under study. This result does not support hypothesis (H2), which states that internal control has no effect on cybersecurity awareness. With the same result, the results showed that awareness of cybersecurity affects the security of accounting information. The results of Table 9 showed that the relationship is positive and statistically significant according to the values (O = 0.438, T = 2.206, P = 0.027). These results confirm that increasing awareness of cybersecurity associated with to improving the protection of accounting systems from potential threats and breaches in the banks under study. These results do not support hypothesis (H3), which states that there is no effect of awareness of cybersecurity on the security of accounting information. The results, as shown in Table 10, regarding the indirect effect, also showed that cybersecurity awareness plays a statistically significant mediating role in the relationship between internal control and accounting information security. The indirect effect value was (O = 0.348, T = 2.105, P = 0.035), indicating that part of the impact of internal control on accounting information security is transmitted through the level of cybersecurity awareness. This result does not support Hypothesis (H4), which states that cybersecurity awareness has no effect on the relationship between internal control and accounting information security.

**Table 8.** Total direct effect.

	Original sample (O)	Sample mean (M)	Standard deviation (STDEV)	T statistics (|O/STDEV|)	P values
Internal control -> AIS	0.348	0.384	0.165	2.105	0.035

**Table 9.** Indirect effects including CAW as mediator

	Original sample (O)	Sample mean (M)	Standard deviation (STDEV)	T statistics (|O/STDEV|)	P values
CAW-> AIS	0.438	0.478	0.199	2.206	0.027
Internal control -> AIS	0.786	0.797	0.041	19.001	0.008
Internal control -> CAW	0.795	0.802	0.043	18.447	0.000

**Table 10.** Specific indirect effect.

	Original sample (O)	Sample mean (M)	Standard deviation (STDEV)	T statistics (|O/STDEV|)	P values
Internal control -> CAW -> AIS	0.348	0.384	0.165	2.105	0.035

The previous results related to the mediating relationship are confirmed in Table 8, where it was shown that the estimated value of the total indirect effect of internal control on accounting information security was statistically significant and positive, reinforcing the hypothesis that cybersecurity awareness is a key factor in the hypothesized model and has a positive effect.

In conclusion, it can be concluded that the structural model shown in Figure 3 has proven highly efficient in explaining the impact relationships between the studied variables. The high values of T and low P indicate that the relationships between the studied variables in the model are not a coincidence, but rather reflect a logical and practical connection between internal control (IC), cybersecurity awareness (CAW), and accounting information security (AIS), in line with modern theoretical trends that confirm that creating a strong internal control environment and raising the level of cybersecurity awareness together help in enhancing the security of accounting information in the studied banking institutions. (See the summary of the hypothesis testing, Table 11).

**Table 11.** Hypothesis testing summary.

Hyp.	Statement	Path tested	Result	Conclusion
**H1**	There is no statistically significant effect of internal control on accounting information security in government banks in Anbar Governorate.	IC → AIS (Direct Effect)	β = 0.786, T = 19.00, p = 0.008 (significant)	Rejected
**H2**	There is no statistically significant effect of internal control on cybersecurity awareness in government banks in Anbar Governorate.	IC → CAW	β = 0.795, T = 18.447, p = 0.000	Rejected
**H3**	There is no statistically significant effect of cybersecurity awareness on accounting information security in government banks in Anbar Governorate.	CAW → AIS	β = 0.438, T = 2.206, p = 0.027	Rejected
**H4**	There is no statistically significant effect of internal control on accounting information security when cybersecurity awareness is present as a mediating variable.	IC → CAW → AIS (Indirect Effect)	Indirect effect = 0.348, T = 2.105, p = 0.035 (Significant positive mediation)	Rejected

## 6. Discussion of results

The results of the statistical analysis showed that the internal control environment in the banks under study enjoys a good level of implementation, as senior management demonstrated a clear interest in control reports and the principle of individual and collective responsibility for control. It also became clear that financial and administrative reports are effectively used as a control tool to help improve performance and identify deviations, reflecting organizational awareness of the importance of control activities in supporting the stability of banking operations. The results also showed that accounting information security is one of the most important elements of accounting system efficiency, as the bank is committed to keeping data confidential, restricting access rights, and maintaining backup copies. This enhances management’s confidence in the accuracy of information and decision-making. Regarding cybersecurity awareness, it was found that employees have a good understanding of the nature of cyber risks and the need to adhere to security measures, such as changing passwords, not sharing them, and examining the sources of emails. However, the results indicated a need for further training courses to enhance the efficiency of employees in this field. In general, the results revealed a complementary relationship between the internal control environment, information security, and cybersecurity awareness, with each contributing to the enhancement of the other. The stronger and more effective the internal control environment, the greater the ability to protect data and information. As employees’ cybersecurity awareness increases, the effectiveness of this control increases, and the quality of the accounting system improves.

## 7. Study limitations

In the interest of transparency, we acknowledge several limitations of this study. First, the study employed a cross-sectional design, which restricted our ability to infer causation. Consequently, the results reflect only correlational relationships without indicating the direction of causal effects. Second, the study comprises a single institution and two branches, which may affect the generalizability of the findings to other research communities. Third, the study data relied on self-reported perceptions and did not test for common methodological bias, despite corrective measures implemented to ensure participant anonymity. Finally, the sample size limitations suggest the need for larger sample sizes in future research on the same topic to validate the findings and ensure their generalizability.

## 8. Conclusion and recommendation

In conclusion, this research, which examined the relationship between the internal control environment, accounting information security, and cybersecurity awareness in banks, sought to clarify the vital role played by effective control systems in enhancing information security and ensuring the integrity of financial and accounting procedures. This topic is particularly important considering the accelerating digital transformation taking place in the banking sector and the accompanying cyber risks that require institutional readiness and high professional awareness among employees.

The research reached a set of important findings that summarize the essence of the objectives achieved. The results of the statistical analysis showed that the internal control environment in the banks studied enjoys an acceptable level of organization and effectiveness, and that senior management pays clear attention to audit reports and the application of the principles of segregation of duties and responsibilities, which positively impacts the stability of internal operations. It also revealed that accounting information security represents a fundamental pillar of the accounting system, as banks implement precise procedures to protect data and its confidentiality and continuously update their security systems. Regarding cybersecurity awareness, the results showed that employees are aware of the importance of protecting electronic systems, despite the need for further training and awareness to address the rapid developments in this field.

Based on these results, it can be said that the research clearly answered the question posed at the outset: to determine the extent to which the internal control environment, information security, and cybersecurity awareness impact the efficiency of the accounting system and the protection of its data. The results demonstrated a complementary relationship between these dimensions, with each contributing to the support of the other. This confirms that effective information protection can only be achieved through a robust control system and an institutional culture based on awareness and responsibility. From the above, several conclusions and recommendations can be drawn, the most important of which is that a strong control system represents the cornerstone of information security, and that developing the technological infrastructure is not sufficient unless accompanied by high human and professional awareness. Therefore, it is recommended to strengthen the role of internal audit units and update their policies periodically. It is also recommended to intensify training programs that enhance employees’ awareness of cyber risks, in addition to encouraging cooperation between audit, risk, and information technology departments to ensure integrated protection of financial systems.

## Data Availability

**Repository Name:** [Study data entitled: Cybersecurity Awareness as a Mediating Variable in the Relationship between ICS and AIS in Iraqi State Banks] available at https://doi.org/10.5281/zenodo.18100387 ( Al-Mohammedi et al., 2025) The project contains the following Underlying data:
-
**[Questionnaire Form.doc]** (This file contains a questionnaire list designed using a five-point Likert scale, including variables, indicators, and items). -
**[data.xlsx]** (This file contains the raw data used in the statistical analysis, which was collected from the target study sample.) Data are available under the terms of the Creative Commons Attribution 4.0 International license (CC-BY 4.0).
