# Supplementary material for: Cybersecurity Awareness as a Mediating Variable in the Relationship between ICS and AIS in Iraqi State Banks
Source: F1000Res. 2026 May 5;15:294. Originally published 2026 Feb 18. [Version 2] doi: 10.12688/f1000research.175421.2 (PMC13084233; doi:10.12688/f1000research.175421.2)
Supplement: Peer review history

# Reviewer report: Reviewer response for version 2

**DOI:** 10.5256/f1000research.199164.r481832

**Published:** 2026-05-13

**Authors:** Hassan Rehan

**Affiliations:** 1 Purdue University, West Lafayette, Indiana, USA

I have reviewed Version 2 of this manuscript and the authors' response to prior reviewer comments. The revisions address several procedural concerns adequately. However, substantive methodological issues remain that affect the validity of the findings.

**What has been adequately addressed:**

 The R² interpretation has been corrected and causal language replaced with associational terminology throughout. The limitations section now transparently acknowledges the cross-sectional design, single-institution scope, sample size constraints, and absence of a dedicated common method bias test. The questionnaire has been embedded as a supplementary appendix. The Zenodo data availability statement has been updated. These are meaningful improvements to the manuscript's transparency.

**Remaining concerns:**

 The sample size of 50 respondents from two branches of a single institution remains a fundamental limitation for PLS-SEM analysis. While the authors acknowledge this in the limitations section, the discussion and conclusion sections still present the findings with a degree of confidence that is not warranted by a sample of this size from a single bank. PLS-SEM with 50 observations and a model of this complexity produces unstable path coefficient estimates. The bootstrapping procedure with 5,000 samples does not compensate for an inadequate original sample — it only resamples what exists. The authors should more explicitly temper their discussion language to reflect this constraint, particularly in Section 6 where findings are discussed as if they reflect general banking sector behavior in Anbar Governorate.

 The common method bias issue has been acknowledged as a limitation but not addressed analytically. Given that all three variables — ICS, CAW, and AIS — were measured from the same respondents in a single survey administration, common method variance is a plausible alternative explanation for the observed associations. The authors state procedural remedies were used but acknowledge no statistical test was performed. At minimum, a Harman single-factor test should be reported, or the authors should more explicitly caveat that observed path coefficients may be inflated by common method variance.

 The HTMT values reported (0.839–0.871) are below the 0.90 threshold but are notably high, approaching the boundary. With a sample of 50, discriminant validity conclusions should be stated cautiously. The manuscript does not acknowledge this proximity to the threshold.

**Recommendation:**

 The revision represents a genuine improvement over Version 1. The core research question is relevant and the analytical approach is appropriate in principle. However, the discussion section needs further tempering to reflect the sample size and common method bias limitations more consistently. Subject to those revisions, I would approve with reservations.

Is the work clearly and accurately presented and does it cite the current literature?

Partly

If applicable, is the statistical analysis and its interpretation appropriate?

Yes

Are all the source data underlying the results available to ensure full reproducibility?

Yes

Is the study design appropriate and is the work technically sound?

Yes

Are the conclusions drawn adequately supported by the results?

Partly

Are sufficient details of methods and analysis provided to allow replication by others?

Partly

Reviewer Expertise:

Machine Learning, Artificial Intelligence, Cloud Computing, Data Engineering, Data Analytics, Deep Learning, Big Data Systems, Distributed Computing, AI-driven Security Systems, Intelligent Data Processing

I confirm that I have read this submission and believe that I have an appropriate level of expertise to confirm that it is of an acceptable scientific standard, however I have significant reservations, as outlined above.

# Reviewer report: Reviewer response for version 2

**DOI:** 10.5256/f1000research.199164.r481833

**Published:** 2026-05-07

**Authors:** Mostafa Rahmany

**Affiliations:** 1 Buckinghamshire New University, High Wycombe, England, UK

**Constructive assessment:**

 This revised version is improved and addresses several of the main concerns raised in the earlier reviews. In particular, the manuscript now includes a limitations section, the data-availability wording has been corrected, the demographic gender mismatch has been removed, and the authors state that the questionnaire instrument has been added as supplementary material. These changes improve transparency and make the paper stronger than version 1. The study remains relevant and potentially useful, and the overall PLS-SEM framework is still acceptable for the research question. However, I do not think the manuscript has fully reached the standard for an unrestricted approval yet, because several important issues remain unresolved.

1. The paper still overstates its scope. The title and several parts of the abstract and conclusion continue to frame the findings as applying to “Iraqi State Banks,” while the actual sample is limited to 50 respondents from two branches of a single bank. The conclusions should therefore remain more tightly tied to the sampled setting.
2. Despite the stated revision, causal wording still appears throughout the manuscript. Terms such as “effect,” “impact,” “contributes,” “enhancing,” and similar formulations are still used repeatedly in ways that go beyond what a cross-sectional self-report design can support. The language should be made consistently associational throughout.
3. Some reporting inconsistencies are still present and should be corrected before full approval. For example, Table 8 is labeled as a “Total direct effect,” but the reported values correspond to the indirect effect and match Table 10. Table 9 is titled as indirect effects, yet it contains the direct paths. There is also still inconsistency between the demographic narrative and Table 1, especially in the discussion of years of experience. In addition, the abbreviation “IC” is still used both for the overall internal control construct and for the “information and communication” dimension, which remains confusing.
4. The English is improved compared with the earlier version, but the manuscript still contains awkward phrasing, grammatical problems, and unclear sentences in several sections. The article is readable, but it still needs another careful language edit.

Overall, I think the manuscript has academic merit and has clearly improved after revision, but the remaining issues are still substantive enough that I recommend **Approved with Reservations**, not full approval.

**In Summary:**

 The paper continues to make a useful, context-specific contribution with competent analytics and open data practices. However, the incomplete response to my first review points (especially language, scope over-generalization, table errors, and missing questionnaire embedding) means it is not yet ready for full indexing. With the targeted revisions listed above, it would become acceptable.

**Questions**

1. **Is the work clearly and accurately presented and does it cite the current literature?**
**Partly **(Improved overall, but some wording and reporting inconsistencies remain).
2. **Is the study design appropriate and does the work have academic merit?**
**Yes **(The design fits the research question and has academic merit).
3. **Are sufficient details of methods and analysis provided to allow replication by others?**
**Partly** (Methods and data are provided, but some details are still not fully clear).
4. **If applicable, is the statistical analysis and its interpretation appropriate?**
**Partly **(The method is suitable, but some interpretation and table presentation remain confusing.).
5. **Are all the source data underlying the results available to ensure full reproducibility?**
**Yes **(The raw data and questionnaire are reported as available in the repository).
6. **Are the conclusions drawn adequately supported by the results?**
**Partly **(Supported within the sample, but still somewhat broader than the data justify).
7. **Approval status:**
**Approved with Reservations**

Is the work clearly and accurately presented and does it cite the current literature?

Partly

If applicable, is the statistical analysis and its interpretation appropriate?

Yes

Are all the source data underlying the results available to ensure full reproducibility?

Yes

Is the study design appropriate and is the work technically sound?

Yes

Are the conclusions drawn adequately supported by the results?

Partly

Are sufficient details of methods and analysis provided to allow replication by others?

Partly

Reviewer Expertise:

Cybersecurity, AI & ML, Network Infrastructure, Network Security, Information Technology, Oil & Gas Industry Management, Oil & Gas Cybersecurity, AI Security, Healthcare Security, Healthcare Cybersecurity Framework,

I confirm that I have read this submission and believe that I have an appropriate level of expertise to confirm that it is of an acceptable scientific standard, however I have significant reservations, as outlined above.

# Reviewer report: Reviewer response for version 1

**DOI:** 10.5256/f1000research.193402.r473915

**Published:** 2026-04-15

**Authors:** Hassan Rehan

**Affiliations:** 1 Purdue University, West Lafayette, Indiana, USA

This study examines cybersecurity awareness as a mediating variable in the relationship between internal control systems (ICS) and accounting information security (AIS) in Iraqi government banks, using PLS-SEM with bootstrapping on a sample of 50 employees from two branches of Al-Rashid Bank in Fallujah and Ramadi. The research question is well-motivated and contextually relevant — the integration of internal control with information security in Arab public-sector banking remains underexplored, and cybersecurity awareness as a mediator is a theoretically defensible and practically meaningful construct to investigate. The conceptual model is grounded in COSO (2013) and supported by a coherent body of literature. The analytical execution — measurement model evaluation, structural model testing, and mediation analysis via specific indirect effects — follows accepted PLS-SEM conventions and is transparently reported. Open deposit of the raw dataset and questionnaire on Zenodo is commendable and enhances reproducibility. 

 Presentation and literature

 The manuscript contains persistent grammatical errors, awkward sentence constructions, and redundant passages throughout the introduction, literature review, and discussion sections. Several sentences are incomplete or shift register mid-paragraph. The R² interpretation in Section 5.1 is inverted — the text states that 68% of variation "in internal control" is explained by "the AIS variable," when the correct reading is that AIS variance is explained by internal control. This error in direction of interpretation recurs for the CAW model and must be corrected. The literature coverage is adequate for the topic and includes recent work, though several references lack full citation details and one source is listed only as "n.d." without a retrieval date.

 Study design and technical soundness 

 The PLS-SEM mediation design is appropriate for the research objectives. The measurement model demonstrates acceptable reliability (Cronbach's α 0.886–0.928, composite reliability >0.90, AVE >0.50 for all constructs) and discriminant validity (HTMT values 0.839–0.871, all below the 0.90 threshold). Outer loadings are all statistically significant and the majority exceed 0.70, with four items (caw1, caw5, caw7, caw8) falling slightly below this threshold but remaining statistically meaningful. VIF values confirm the absence of problematic multicollinearity. Bootstrapping with 5,000 samples is appropriate for indirect effect estimation.

 Replication details 

 The questionnaire items are not reproduced in the manuscript body, only referenced via Zenodo. While the Zenodo deposit makes them accessible, embedding the full instrument as a supplementary appendix would substantially improve transparency and immediate replicability. The demographic table (Table 1) omits a gender column despite the text explicitly stating that most respondents are male — this must be corrected either by adding the column or removing the claim.

 Statistical interpretation 

 Path analysis results are correctly reported and interpreted. All four hypotheses are rejected at p < 0.05, supporting positive direct effects of IC on AIS (β = 0.786, T = 19.001) and on CAW (β = 0.795, T = 18.447), a positive effect of CAW on AIS (β = 0.438, T = 2.206), and a significant indirect effect through the mediator (β = 0.348, T = 2.105, p = 0.035). One reporting error requires correction: Tables 8 and 9 both carry the heading "Total indirect effect" — Table 9 should be headed "Direct effects" to reflect its content accurately.

 Source data 

 Raw data and the questionnaire are openly deposited on Zenodo. The data availability statement contains a mismatch — it describes the deposit as concerning "Industrial Control Systems and Management Information Systems" while the actual Zenodo record uses "ICS and AIS." This must be corrected for consistency.

 Support for conclusions 

 The core finding — that cybersecurity awareness partially mediates the relationship between internal control and accounting information security — is statistically supported within the study's scope. However, the manuscript draws conclusions about "Iraqi State Banks" and "government banks in Anbar Governorate" that substantially exceed what a convenience sample of 50 respondents from two branches of a single institution can credibly support. The title, abstract, and conclusion sections must be revised to accurately reflect the narrow institutional scope of the sample. Common-method bias is a legitimate concern in a single-source, self-report survey design and has not been tested — at minimum, a Harman's single-factor test or full collinearity VIF assessment should be reported and the limitation acknowledged explicitly. The high path coefficients (β = 0.786–0.795) combined with the small sample also warrant a caution regarding potential overfitting. Throughout the manuscript, causal language — "contributes to," "enhances," "leads to" — should be tempered to associational language consistent with the cross-sectional design.

 Required changes before approval:

 The R² directional interpretation in Section 5.1 must be corrected. The gender column must be added to Table 1 or the demographic claim removed. The duplicate table heading ("Total indirect effect" on both Tables 8 and 9) must be corrected. The data availability statement Zenodo description must be corrected to match the actual deposit title. The title, abstract, and conclusions must be revised to reflect the single-institution, two-branch scope of the sample. Causal language throughout must be softened to associational. A common-method bias test should be conducted and reported, or its absence explicitly justified and acknowledged as a limitation. A dedicated limitations section should be added covering sample size, single-institution scope, cross-sectional design, self-report bias, and the absence of common-method bias testing. The full questionnaire instrument should be embedded as a supplementary appendix. Professional English language editing is required throughout.

Is the work clearly and accurately presented and does it cite the current literature?

Partly

If applicable, is the statistical analysis and its interpretation appropriate?

Yes

Are all the source data underlying the results available to ensure full reproducibility?

Yes

Is the study design appropriate and is the work technically sound?

Yes

Are the conclusions drawn adequately supported by the results?

Partly

Are sufficient details of methods and analysis provided to allow replication by others?

Partly

Reviewer Expertise:

Machine Learning, Artificial Intelligence, Cloud Computing, Data Engineering, Data Analytics, Deep Learning, Big Data Systems, Distributed Computing, AI-driven Security Systems, Intelligent Data Processing

I confirm that I have read this submission and believe that I have an appropriate level of expertise to confirm that it is of an acceptable scientific standard, however I have significant reservations, as outlined above.

# Reviewer report: Reviewer response for version 1

**DOI:** 10.5256/f1000research.193402.r473923

**Published:** 2026-04-10

**Authors:** Mostafa Rahmany

**Affiliations:** 1 Buckinghamshire New University, High Wycombe, England, UK

***Constructive assessment:***

 This is a timely and relevant study that addresses an important gap in the accounting and cybersecurity literature, particularly in the under-researched context of Iraqi government banks. The authors correctly identify cybersecurity awareness as a potential mediator between internal control systems (ICS) and accounting information security (AIS). They develop a clear conceptual model based on COSO and relevant literature, derive four testable hypotheses, and apply PLS-SEM appropriately to test direct and indirect effects. The measurement model shows strong reliability (Cronbach’s α 0.886–0.928, composite reliability >0.90, AVE >0.50) and discriminant validity (HTMT <0.90). Path coefficients are large and statistically significant, supporting the rejection of all null hypotheses and demonstrating a positive mediating role for cybersecurity awareness. Open sharing of the raw dataset and questionnaire on Zenodo is a major strength and promotes reproducibility.

 However, several limitations require attention before the work can be considered fully robust for indexing:

**1. Sample size and generalizability (major concern):** The study uses N=50 respondents from only two branches of a single institution (Al-Rashid Bank in Fallujah and Ramadi). This is a convenience sample that cannot credibly support conclusions about “Iraqi State Banks” or even “government banks in Anbar Governorate.” While PLS-SEM can function with small samples, the explanatory power claims (R² ≈ 0.68 for AIS) and very high path coefficients (β = 0.786–0.795) warrant caution regarding statistical power, overfitting, and common-method bias (not tested).

**2. Language and presentation:** The manuscript contains numerous grammatical errors, awkward phrasing, sentence fragments, and repetitions that reduce clarity and readability (especially in the introduction, literature review, and discussion). Professional English editing is essential.

**3. Minor technical and reporting issues:**

**3.1** Demographic Table 1 description mentions “most of the sample members are male,” yet the table does not include a gender column.

**3.2** Table 8 and Table 9 both carry the heading “Total indirect effect” (copy-paste error).

**3.3** The data-availability statement incorrectly describes the Zenodo deposit as concerning “Industrial Control Systems and Management Information Systems.” The actual Zenodo title correctly uses “ICS and AIS” (matching the article). This mismatch must be corrected.

**3.4** Questionnaire items are not reproduced in the manuscript (only referenced to the Zenodo file), which slightly hinders immediate replication.

**3.5** Causal language (“contribute to,” “enhancing,” “positive role”) should be tempered to “associated with” or “predicts” given the cross-sectional, self-report design.

**4. Limitations section:** Currently minimal. A fuller discussion of sample limitations, self-report bias, single-institution scope, and cross-sectional nature is needed.

 Aspects I have not been able to assess: I did not download and re-run the raw .xlsx file from Zenodo to independently verify every bootstrap result or check for data-entry anomalies (though the reported statistics appear internally consistent and the deposit is openly accessible).

**5. Recommendations to authors**

**5.1 **Undertake a full professional language edit. 

**5.2 **Explicitly acknowledge sample limitations and revise title/abstract/conclusions to reflect the narrow scope (e.g., “in selected branches of Al-Rashid Bank, Anbar Governorate”). 

**5.3** Correct the Zenodo reference and, ideally, embed the full questionnaire items (or a supplementary file) in the next version. 

**5.4** Expand the limitations and future-research sections. 

**5.5** Consider testing for common-method bias (e.g., Harman’s single-factor test or full collinearity VIF). 

 Add a brief practical implications subsection for Iraqi banking regulators or internal-audit units.

 These revisions are feasible and would substantially strengthen the paper.

 6. Answers to mandatory reviewer questions (Research Article)

 1-Is the work clearly and accurately presented, and does it cite the current literature? 

 Partly. The literature is relevant and up to date, but language/grammar issues and the Zenodo title mismatch reduce clarity and accuracy.

 2. Is the study design appropriate and is the work technically sound? 

 Yes (the PLS-SEM mediation design and execution are technically sound), but the extremely narrow convenience sample limits academic merit and generalizability.

 3. Are sufficient details of methods and analysis provided to allow replication by others? 

 Partly, while mostly (full dataset and questionnaire are openly available; PLS-SEM parameters are reported in detail), but embedding the questionnaire items would improve transparency.

 4- If applicable, is the statistical analysis and its interpretation appropriate? 

 Yes. Standard PLS-SEM procedures were followed and correctly interpreted.

 5- Are all the source data underlying the results available to ensure full reproducibility?

 Yes (via the Zenodo repository).

 6-Are the conclusions drawn adequately supported by the results? 

 Partly, within the study’s narrow scope, however, the authors over-generalize beyond the sampled branches.

**Approval status:**

 Approved with reservations

**In Summary:**

 The paper makes a useful, context-specific contribution and the analytical work is competently executed, but the small/single-bank sample, language problems, and minor reporting inaccuracies prevent full approval in its current form. With the recommended revisions, it would be suitable for indexing.

Is the work clearly and accurately presented and does it cite the current literature?

Partly

If applicable, is the statistical analysis and its interpretation appropriate?

Yes

Are all the source data underlying the results available to ensure full reproducibility?

Yes

Is the study design appropriate and is the work technically sound?

Yes

Are the conclusions drawn adequately supported by the results?

Partly

Are sufficient details of methods and analysis provided to allow replication by others?

Partly

Reviewer Expertise:

Cybersecurity, AI & ML, Network Infrastructure, Network Security, Information Technology, Oil & Gas Industry Management, Oil & Gas Cybersecurity, AI Security, Healthcare Security, Healthcare Cybersecurity Framework,

I confirm that I have read this submission and believe that I have an appropriate level of expertise to confirm that it is of an acceptable scientific standard, however I have significant reservations, as outlined above.

# Reviewer report: Reviewer response for version 1

**DOI:** 10.5256/f1000research.193402.r465406

**Published:** 2026-03-23

**Authors:** Muthana R. Jasim

**Affiliations:** 1 College of Administration and Economics - Department of Accounting, Tikrit University, Tikrit, Saladin Governorate, Iraq

The manuscript addresses an important and timely topic—the role of cybersecurity awareness as a mediator between internal control systems and accounting information security in public banks—and uses PLS-SEM to test theoretically sensible hypotheses. Strengths include a clear theoretical framing, transparent reporting of reliability/validity measures, and open sharing of the underlying dataset. However, the study’s generalizability and causal inference are limited by the small and convenience-based sample (N=50 from two branches of a single bank). I recommend the authors temper causal language, more explicitly discuss limitations related to sample size and potential self-report bias and include the survey instrument items in a manuscript appendix (or cite the Zenodo file directly).  Overall, the paper makes a useful contribution but would benefit from these clarifications before indexing.

Is the work clearly and accurately presented and does it cite the current literature?

Yes

If applicable, is the statistical analysis and its interpretation appropriate?

Yes

Are all the source data underlying the results available to ensure full reproducibility?

Partly

Is the study design appropriate and is the work technically sound?

Yes

Are the conclusions drawn adequately supported by the results?

Yes

Are sufficient details of methods and analysis provided to allow replication by others?

Yes

Reviewer Expertise:

Financial AccountantInternational Financial Reporting Standards

I confirm that I have read this submission and believe that I have an appropriate level of expertise to confirm that it is of an acceptable scientific standard.
